# An Improved Transplantation Strategy for Mouse Mesenchymal Stem Cells in an Acute Myocardial Infarction Model

**DOI:** 10.1371/journal.pone.0021005

**Published:** 2011-06-17

**Authors:** Jianliang Jin, Yingming Zhao, Xiao Tan, Chun Guo, Zhijian Yang, Dengshun Miao

**Affiliations:** 1 The Research Center for Bone and Stem Cells, Department of Anatomy, Histology and Embryology, Nanjing Medical University, Nanjing, Jiangsu, The People's Republic of China; 2 Department of Cardiology, The First Affiliated Hospital, Nanjing Medical University, Nanjing, The People's Republic of China; University of South Florida, United States of America

## Abstract

To develop an effective therapeutic strategy for cardiac regeneration using bone marrow mesenchymal stem cells (BM-MSCs), the primary mouse BM-MSCs (1^st^ BM-MSCs) and 5^th^ passage BM-MSCs from β-galactosidase transgenic mice were respectively intramyocardially transplanted into the acute myocardial infarction (AMI) model of wild type mice. At the 6^th^ week, animals/tissues from the 1^st^ BM-MSCs group, the 5^th^ passage BM-MSCs group, control group were examined. Our results revealed that, compared to the 5^th^ passage BM-MSCs, the 1^st^ BM-MSCs had better therapeutic effects in the mouse MI model. The 1^st^ BM-MSCs maintained greater differentiation potentials towards cardiomocytes or vascular endothelial cells *in vitro*. This is indicated by higher expressions of cardiomyocyte and vascular endothelial cell mature markers *in vitro*. Furthermore, we identified that 24 proteins were down-regulated and 3 proteins were up-regulated in the 5^th^ BM-MSCs in comparison to the 1^st^ BM-MSCs, using mass spectrometry following two-dimensional electrophoresis. Our data suggest that transplantation of the 1^st^ BM-MSCs may be an effective therapeutic strategy for cardiac tissue regeneration following AMI, and altered protein expression profiles between the 1^st^ BM-MSCs and 5^th^ passage BM-MSCs may account for the difference in their maintenance of stemness and their therapeutic effects following AMI.

## Introduction

Acute myocardial infarction (AMI), a main presentation of ischemic heart disease, is one leading cause of death globally. Although pharmacologic intervention and coronary artery bypass grafting, which can restore the blood flow (reperfusion), keep viable myocardium working, these approaches cannot rescue dying myocardium or restore normal cardiac functions. Moreover, new therapeutic approaches are needed for patients under critical conditions suffering from small vessel diffuse lesions, who are unable to withstand coronary angioplasty and for whom medication is not effective. Therefore, it is imperative to develop an effective therapeutic approach to regenerating myocardium and restoring its normal contractile-relaxant function following AMI. For a long time, it was commonly accepted that cardiomyocytes, as terminally differentiated cell type, could not be regenerated. Recently, accumulated evidence indicates that cardiac muscles can be regenerated or repaired via a variety of ways including stem cell technology [1].

To use stem cell technology for the treatment of AMI has been one of the hot areas in cardiovascular research. BM-MSCs are readily obtainable, safe, ethical, and an easy handling resource for tissue transplantation with a high proliferation/differentiation potential. It is low risk for immunorejection and pathogen transmission. It also has a stable genetic background, so that has been used as seeding cells in tissue engineering and stem cell therapy [2], [3], [4], [5], [6]. BM-MSCs are primary seeding cells of heart transplantation used for the treatment of AMI. Accumulated evidence indicates that the transplantation of BM-MSCs can effectively prevent ventricular remodeling, improve cardiac performance, and ameliorate the outcome of ST-elevated AMI [7], [8], [9], [10], [11], [12].

Passaging *in vitro* has been a conventional approach to obtaining a large number of BM-MSCs as required by transplantation. However, during the sub-culture processing, BM-MSCs gradually lose their differentiation potency towards cardiomyocytes and vascular endothelial cells, and this is likely to result in less satisfactory improvement in cardiac performance [13]. Previous studies suggest that biological properties of BM-MSCs are not everlasting features and their proliferation and differentiation properties decline along with passaging process [13], [14]. How to obtain sufficient number of seeding cells, with a good quality of stem cell biological properties for transplantation, remains the center of stem cell therapy research.

Although therapy based on BM-MSCs has been gradually introduced into clinics [15], their basic biological characteristics remain largely unknown. It is generally accepted that BM-MSCs are a highly adhesive fibroblast-like cell type. Our previous results [16] jointly with data from others [17] indicate the existence of a population of non-adherent, small, round cells, with self-renewal and multilineage differentiation potential in adult bone marrow, and those cells are capable of forming CFU-Fs *in vitro*. Accordingly we have developed the “Pour-off” method to amplify the 1^st^ BM-MSC.

It has been suggested that bone marrow milieu can preserve stem cell properties of BM-MSCs [18]. Main marrow components including hematopoietic cell population are present in primary culture of BM-MSCs, but those components are missing when BM-MSCs undergo repeat passaging on tissue culture plastic. This change accounts for major differences between primary BM-MSCs and repeat passage BM-MSCs in terms of culture conditions. It is likely that hematopoietic cells maintain the self-renewal and differentiation potency of the 1^st^ BM-MSCs by releasing cytokines and growth factors. Therefore, depletion of hematopoietic cells may result in decline of differentiation potency of repeat passage BM-MSCs towards cardiomyocytes and vascular endothelial cells. Presumably the “Pour-off” method not only can ensure to prepare sufficient numbers of BM-MSCs for the transplantation, but also can help to retain differentiation potency of BM-MSCs, possibly through the actions of hematopoietic cells, which are present in conditioned culture media. Therefore, we hypothesized that: i) the 1^st^ BM-MSCs amplified using the “Pour-off” method would be superior to repeat passage BM-MSCs in terms of the potency of proliferation and differentiation, and paracrine effects, and ii) the transplantation of the 1^st^ BM-MSCs would be superior to that of repeat passage BM-MSCs in terms of therapeutic effectiveness for AMI. In the present study, the primary mouse BM-MSCs (1^st^ BM-MSCs) and 5^th^ passage BM-MSCs from β-galactosidase transgenic mice were respectively intramyocardially transplanted into AMI model of wild type mice. At the 6^th^ week, animals/tissues from the 1^st^ BM-MSCs group, the 5^th^ passage BM-MSCs group, control group were examined. To investigate the potential mechanisms underlying the differential therapeutic effects, we utilized *in vitro* culture systems and cellular/molecular and proteomics techniques. We examined the profiles of proliferation, apoptosis, and differentiation potentials towards cardiomyocytes and vascular endothelial cells of the 1^st^ or the 5th passage BM-MSCs, and expressions of gene markers for pluripotent stem cells or tissue committed stem cells expressed by the two types of cells, and identified protein expression profiles between the 1^st^ BM-MSCs and the 5th passage BM-MSCs.

## Materials and Methods

Adult C57BL/6J and β-gal transgenic mice weighing 20±2 g were obtained from the Jackson Laboratory. The use of animals in this study was approved by the Institutional Animal Care and Use Committee of Nanjing Medical University (Approval ID 2008-00318).

### BM-MSC cultures and harvesting

β-gal transgene mice were killed by cervical dislocation. Total BMCs were flushed out of tibias and femurs. After washing, cells were centrifuged, and resuspended in 10 ml normal culture medium consisting of α-MEM containing 10% (v/v) fetal bovine serum, 2 mM L-Glutamine and 50 µg/ml Ascorbic acid, to a final concentration of 10^7^ viable cells in 10 cm petri dishes and kept in a humidified 5% CO_2_ incubator at 37°C. “Pour-off” cultures were performed as previously described [16]. Briefly, after total BMCs were kept in a humidified 5% CO_2_ incubator at 37°C for 24 h in the absence or presence of 10^−8^ M 1,25-dihydroxyvitamin D_3_, non-adherent cells (NA) were resuspended into a new 10 cm petri dish and repeated 4 times again in this way. Fresh medium was added to all the dishes. Adherent BMC cells obtained from either way were termed as the 1^st^ BM-MSCs. Subsequently, confluent 1^st^ BM-MSCs by “Pouring-off” were trypsined and sub-cultured. The 1^st^ BM-MSCs and passage 5 BM-MSCs were used in the study.

### Lacz staining for β-galactosidase activity

Pre-embedding LacZ staining was performed following a modified version of a previously described method [19].

### Myocardial infarction model and BM-MSCs transplantation

When the Left ventricular anterior transmural MI of 45 mice was established by permanent ligation of the ramus descendens anterior arteriae coronariae sinistrae with silk ligature using C57BL/6J wild type mice. The 1^st^ BM-MSCs group (MI+1^st^ MSCs group) and the 5^th^ BM-MSCs group (MI+5^th^ BM-MSCs group) were separately injected into the border zone surrounding the infarct anteriorly and laterally (total 5.0×10^6^ cells in 0.05 ml α-MEM) with a 31-gauge needle after the ligation of the left anterior descending artery. Control groups including 15 mice were established by injection of the same volume of α-MEM into the infarcted heart (MI group). A sham group was also included in which the surgery was performed but without ligation of the coronary artery (Sham group). MI group and Sham group were detected electrocardiographs using RM6240 system (Chengdu Instrument Company, Chengdu, China) to determine MI models.

### RNA isolation and real-time RT-PCR

RNA was isolated from MSCs using Trizol reagent (Invitrogen Inc., Carlsbad, CA, USA) according to the manufacturer's protocol. Sample mRNA levels were quantified by real-time RT-PCR as previously described [20]. The PCR primers are shown in Table 1.

**Table 1:** Sequences of primers employed for Real time RT-PCR.

Name	S/AS	Sequence	Tm(°C)	bp
Desmin	S	5′-GTGGATGCAGCCACTCTAGC -3′	60	218
	AS	5′-TTAGCCGCGATGGTCTCATAC-3′		
Troponin I	S	5′-TCTGCCAACTACCGAGCCTAT-3′	60	135
	AS	5′-CTCTTCTGCCTCTCGTTCCAT-3′		
MHC-β	S	5′-TTCATCCGAATCCATTTTGGGG-3′	60	194
	AS	5′-GCATAATCGTAGGGGTTGTTGG-3′		
VEGF	S	5′-GGAGATCCTTCGAGGAGCACTT-3′	55	129
	AS	5′-GGCGATTTAGCAGCAGATATAAGAA-3′		
FLT-1	S	5′-GAGGAGGATGAGGGTGTCTATAGGT-3′	55	116
	AS	5′-GTGATCAGCTCCAGGTTTGACTT-3′		
KDR	S	5′-GCCCTGCTGTGGTCTCACTAC-3′	55	97
	AS	5′-CAAAGCATTGCCCATTCGAT-3′		
Oct4	S	5′-CGCCCGCATACGAGTTCT-3′	51	188
	AS	5′-CTTCTCCAACTTCACGGCATT-3′		
CXCR4	S	5′-GACGGACAAGTACCGGCTGC-3′	51	480
	AS	5′-GACAGCTTAGAGATGATGAT-3′		
MET-R	S	5′-CGCGTCGACTTATTCATGG-3′	51	363
	AS	5′-CACACATTGATTGTGGCACC-3′		
LIF-R	S	5′-CCAAGGACGGAACCAGTAGCA-3′	53	153
	AS	5′-GGAGAAATGAGGCGAGTCAA-3′		
Dppa3	S	5′-GCAGTCTACGGAACCGCATT-3′	50	71
	AS	5′-TTGAACTTCCCTCCGGATTTT-3′		
Nanog	S	5′-CGTTCCCAGAATTCGATGCTT-3′	50	106
	AS	5′-TTTTCAGAAATCCCTTCCCTCG-3′		
Rex-1	S	5′-AGATGGCTTCCCTGACGGATA-3′	49	104
	AS	5′-CCTCCAAGCTTTCGAAGGATTT-3′		
RIF-1	S	5′-TTTCCTTGCCCTCTATGA-3′	51	146
	AS	5′-AACAATTTCTCCCAATAGCTT-3′		
Csx	S	5′-AGACCCTCGGGCGGATAAA-3′	48	155
	AS	5′-GCCGCTCCAGCTCGTAGACCT-3′		
GATA-4	S	5′-TCCAGTGCTGTCTGCTCTAAGC-3′	50	266
	AS	5′-TGGCCTGCGATGTCTGAGT-3′		
VE-cadherin	S	5′-TTCAAGCTGCCAGAAAACCA-3′	49	68
	AS	5′-GAGCCTTGTCAGGGTCTTTGG-3′		
MT1-MMP	S	5′-GGACAGCGAGTACCCTA-3′	50	113
	AS	5′-ATTTGTTTCCCTTGTAGAAGT-3′		
MMP2	S	5′-ATCTTTGCAGGAGACAAGTTC-3′	51	114
	AS	5′-GGCATCCAGGTTATCAG-3′		
MMP9	S	5′-TCTACAGAGTCTTTGAGTCCG-3′	53	134
	AS	5′-GGGCTTCCTCTATGATTCAG-3′		
GAPDH	S	5′-CATTTCACTCAAGGTTGTCAGC-3′	55	346
	AS	5′-ATCATACTTGGCAGGTTTCTCC-3′		

Real-time RT-PCR primers used with their name, orientation (S, sense; AS, antisense), sequence, annealing temperature (Tm), and length of amplicon (bp).

### Immunohistochemiscal staining and immunofluorescence labeling procedures

For immunohistochenmistry, the mice were anesthetized with 3% pentobarbital sodium (40 mg/kg) after 6 weeks following transplantation. Myocardial specimens were perfused with 100 ml normal sodium and then perfused and fixed with PLP solution. The 0.5 cm transverse sections of the short axis of the left ventricles below the artery ligation site were removed and embedded in paraffin and cut into 5-µm sections. Serial paraffin sections were deparaffinized, dehydrated, and, for antigen retrieval, steamed for 20 minutes in PBS (0.01 mM pH 7.4) followed by blocking of endogenous peroxidase (3% H_2_O_2_) and preincubation with serum. Primary antibodies against β-galactosidase (Dako Cytomation, Denmark), vascular endothelial growth factor (VEGF) (R&D, America), platelet endothelial cell adherence molecular (PECAM) (Santa Cruz Biotechnology, America), proliferating cell nuclear antigen (PCNA) (Dako Cytomation, Denmark), Troponin I (Santa Cruz Biotechnology) and Desmin (Thermo, America) were used. After washing steps, the sections were incubated with secondary antibody (biotinylated IgG; Sigma), washed again, and processed using the Vectastain ABC-HRP kit (Vector Laboratories, Inc.). The sections were then counterstained with Hematoxylin and mounted with Biomount medium. For immunofluorescence labeling procedures, corresponding affinity-purified Rhodamine (TRITC), Texas Red (TXRD) or FITC-conjugated secondary antibody (Santa Cruz Biotechnology) were used. Nuclei were labeled by DAPI (Sigma) and mounted with medium which prevents quenching of fluorescence (Vector Laboratories, Inc.). After immunohistochemical and immunofluorescence staining, images of the infarct border zone at the posterior left ventricles from a single section were digitally recorded using a rectangular template and three different fields at ×200 magnification. All images were taken with a Sony digital camera, and processed using Northern Eclipse image analysis software, version 5.0 (Empix Imaging, Inc., Mississauga, Canada). The immunopositive area or positive cell numbers from each section were averaged and expressed as the ultimate positive area or the number of positive cells per field.

### TUNEL Staining

Dewaxed paraffin sections were stained with an In Situ Cell Death Detection Kit (Roche Diagnostics Corp.) using a previously described protocol [21].

### Fibrosis quantification and measurement of infarct zone wall thickness

Total collagen was detected in paraffin sections using a method previously described [20]. Six anterolateral sections from each heart were evaluated in their entirety and quantified. The results were expressed as a percentage of each ventricular section. For measurement of infarct zone wall thickness on each section, two measurements were obtained in the peri-infarct region, one on each side of the infarct and averaged. Measurements were obtained in a group-blinded manner [22].

### Echocardiography

Echocardiography was done after ligation of the left anterior descending artery and immediate transplantation of BM-MSCs 1 and 6 weeks later with a 7.5-MHz high-frequency liner phased-array transducer (GE vivid7). Animals were lightly sedated with ketamine (50 mg/kg) for each echocardiogram. Measurements were made by two independent observers unaware of the treatment condition, offline with Philips Qlabz. For analysis of left ventricular function, left ventricular internal diameter at end-diastole (LVIDd), left ventricular internal diameter at end-systole (LVIDs), left ventricular end-diastolic volume(LVEDV) and left ventricular end-systole volume (LVESV) were measured at the anterior wall, from the short-axis view, just below the level of the papillary muscle. The shortening fraction was calculated as (LVEDD-LVESD) 100%/LVEDD, and ejection fraction was measured as (LVEDV-LVESV) 100%/LVEDV.

### Western Blot

Samples of the infarct border zone of the posterior left ventricles from 0.5 cm transverse sections of the short axis below the ligation site were dissected and immediately placed into a lysis buffer containing a cocktail of proteinase inhibitors for protein extraction. Protein extracts from each group were boiled for 5 min in the sample buffer, fractionated by SDS gel electrophoresis and transferred to nitrocellulose membranes. The membrane was blocked for 2 h at 37°C with 5% non-fat dry milk in PBS/Tween 20. The blots were incubated overnight at 4°C with antibodies against Akt (Santa Cruz Biotechnology), phospho-Akt (pAkt) (Santa Cruz Biotechnology), STAT5 (Sigma), phospho-STAT5 (pSTAT5) (Sigma), Pro-Caspase3 (Santa Cruz Biotechnology), Activate-Caspase3 (Cell Signaling Technology) and β-tubulin (Santa Cruz Biotechnology). This was followed by incubation for 1 h with HRP-conjugated secondary antibody. Immunoreactive bands were visualized using enhanced chemiluminescence reagent treatment and exposure to hyperfilm-ECL. The intensity of the bands was measured using Image J version 1.29.

### 2-D Electrophoresis and Mass Spectrometry

Immobilized pH gradient strips (GE Healthcare, San Francisco, CA) were rehydrated using 120 µg of cell total proteins from BM-MSCs. After isoelectric focusing, the strips were equilibrated, run in the Ettan DALT-6 electrophoresis system, and visualized by silver staining, as described previously [23].

The stained gels were scanned, and the Image Master 2D Platinum Software (Version 6.0; GE Healthcare, Swiss Institute of Bioinformatics) was used for spot detection, quantification, and comparative and statistical analyses, as previously described [23]. In each experiment, protein expression in the 1^st^ BM-MSCs and the 5^th^ BM-MSCs were compared at baseline. The values from each group were pooled for the calculation of the means and their respective standard deviations, and independent t-test was performed to determine the significance of the differences between protein expression in the 1^st^ BM-MSCs and the 5^th^ BM-MSCs. Values of P<0.05 were considered as statistically significant.

Differential silver-stained protein spots were excised, dehydrated in acetonitrile, and dried at room temperature. The proteins were reduced with 10 mM DTT and 25 mM NH_4_HCO_3_ at 56°C for 1 h and alkylated in the dark with 55 mM iodoacetamide and 25 mM NH_4_HCO_3_ at room temperature for 45 min in situ. The gel pieces were then thoroughly washed with 25 mM NH_4_HCO_3_, 50% acetonitrile, and 100% acetonitrile in succession and were completely dried in a Speedvac. The dried gel pieces were reswollen with 2–3 µl of trypsin solution (trypsin at a concentration of 10 ng/µl in 25 mM NH_4_HCO_3_). After incubation at 4°C for 30 min, the excess liquid was discarded, and the gel plugs were incubated at 37°C for 12 h. Finally, 0.1% trifluoroacetic acid was added to stop the digestion reaction. The extracted peptide mixture was then analyzed by MALDI-TOF (Biflex, Bruker Daltonics).

Database Searches: Each acquired mass spectrum (m/z range, 700–4000; resolution, 15 000–20 000) was processed using the software Flex Analysis version 2.0 (Bruker Daltonics). The tryptic autodigestion ion peaks (842.51 and 1045.56 Da,) were used as internal standards to validate the external calibration procedure. Matrix and/or autoproteolytic trypsin fragments or known contaminants were removed. The resulting peptide mass lists were first used to search the Swiss-Prot 48.7 database (release 10/09/05; 204 086 sequences; 74,182,688 residues) for Homo sapiens (13,344 sequences) by using Mascot (version 2.1.03) in the automated mode. In cases where we were unable to obtain a confident result, the mass lists were used to further search the TrEMBL 31.7 database (release 10/09/05; 2,506,886 sequences; 805,901,253 residues) for Homo sapiens (58,416 sequences), which is a supplement to the Swiss-Prot database. The search conditions used were as described in previous work [23]. The peptide mass was compared with the theoretical peptide masses of all available proteins from a species. The search conditions used were as follows: 100 ppm for external calibration, one missed cleavage allowed, and modification of cysteines by iodoacetamide, methionine oxidation, and N-terminal pyroglutamylation as variable modifications. The minimal requirement for an identity assignment was 4 matching peptides, and we only considered mouse proteins. The algorithm used for determining the probability of a false-positive match with a given MS spectrum is described elsewhere [24].

### Statistical analysis

Statistical analysis was performed with SPSS for Windows (version 13.0). All datas were described as mean ± S.E.M of determinations in five animals of each group. To analyze the data statistically, we performed Student's t-test and one way ANOVA. Values of P<0.05 were considered statistically significant.

## Results

### Establishment of acute myocardium infarction mouse model

**Figure 1:**
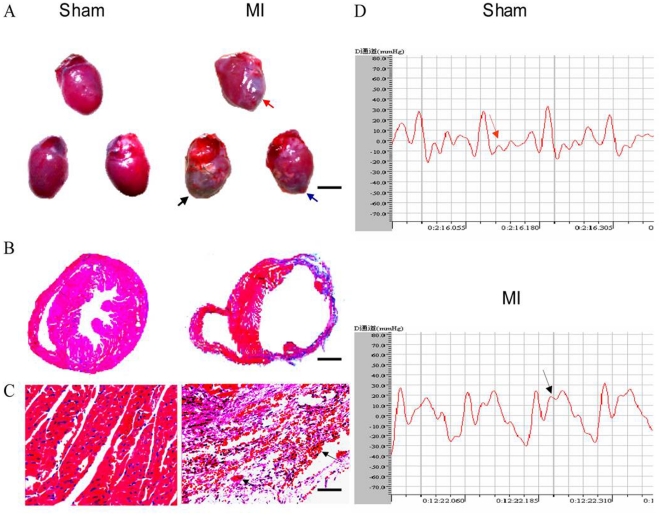
Establishment of acute myocardium infarction mouse model. (A) Representative graphs of the overall hearts from sham operation (left panel) and from the permanent ligation of the ramus descendens anterior arteriae coronariae sinistrae with silk ligature (MI, right panel), red, black and blue arrow indicate for MI occurred in anterior wall, lateral wall and posterior wall of left ventricle, respectively. (B) Representative micrographs of cross sections of ventricles stained with H & E. (C) Representatice micrographs of sections of left ventricles stained with H & E. Black arrows indicate sarcoplasmic condensation occurred in MI areas. Scale bars in A, B and C represent 4000, 800 and 25 µm, respectively. (D) Representative electrocardiographs. The arrow noted that ST segment was elevated in MI animals. Each mini-panel standed for 0.025 s.

An acute myocardium infarction model was established using 57BL/6J mice in reference to a protocol described previously [25]. MI areas were visualized in the anterior wall, lateral wall or posterior wall of the left ventricle respectively as early as 24 hours following the ligation of the left coronary artery (Figure 1A). The left ventricle MI walls became much thinner in comparison to sham controls. Moreover, at the acute stage of injury type MI,inflammatory cell infiltration and representative pathological characteristics of sarcoplasmic condensation were observed (Figures 1B and C). Furthermore, electrocardiographic analysis showed an elevated ST segment in MI mice (Figure 1D).

### Preparation and characterization of seeding cells for the transplantation

**Figure 2:**
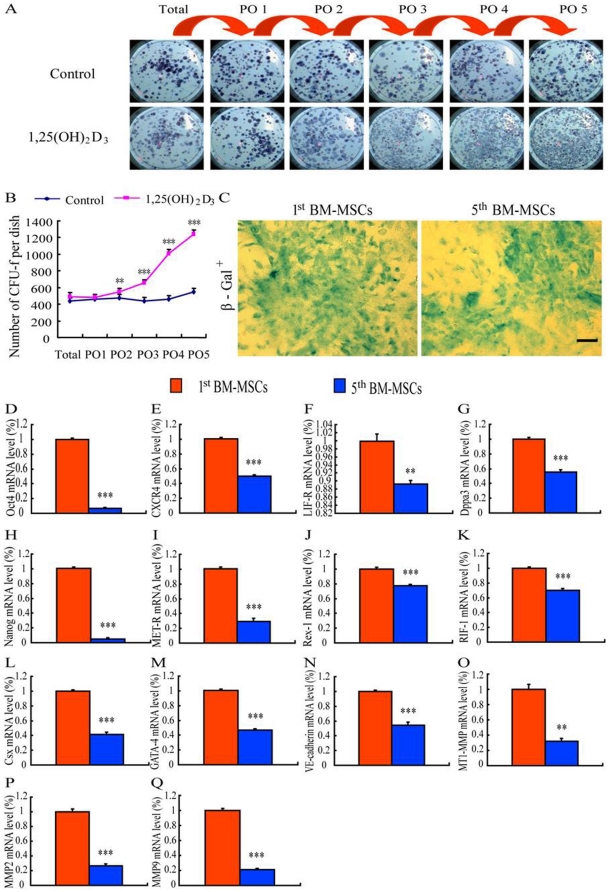
Preparation and characterization of donor cells. (A) Representative methylene blue stained cultures from mouse total BM cells (total BMC), the first pour-off (PO1) (that was, colonies from non-adherent supernatant cells in the first pour-off that became adherent and proliferated), the second pour-off (PO2), the third pour-off (PO3), the fourth pour-off (PO4) and the fifth pour-off (PO5) in the absence (control, upper panel) and presence of 10^−8^ M 1,25(OH)_2_D_3_ (lower panel). The initial red arrow indicated that PO1 was derived from non-adherent supernatant cells of total unmanipulated bone marrow and subsequent red arrows indicated that sequential pour-offs were derived from cultures of previous supernatant cells. (B) The number of CFU-fs was quantitated in the pour-off cultures stained with methylene blue and was depicted as mean±s.e.m. of triplicate determinations. (C) Micrographs of cells from the primary pour off cultures and 5th passaged cultures stained cytochemically for ß-gal activity. Scale bar represents 25 µm. (D) OCT-4, (E) CXCR4, (F) LIF-R, (G) Dppa3, (H) Nanog, (I) MET-R, (J) Rex-1, (K) Rif-1, (L) Csx, (M) GATA-4, (N) VE-cadherin, (O) MT1-MMP, (P) MMP2 and (Q) MMP9 mRNA levels were examined in BM-MSCs from the primary pour-off cultures (1^st^ BM-MSCs) and the 5^th^ passaged cultures (5^th^ BM-MSCs) by real-time RT-PCR. The mRNA levels were calculated as a ratio to the GAPDH mRNA level and expressed relative to levels of the 1^st^ BM-MSCs. Each value was the means±S.E.M. of 5 determinations. **, P<0.01; ***, P<0.001 compared with 1^st^ BM-MSCs. ##, P<0.01; ###, P<0.001 compared with control cultures.

We have showed previously that not only total BMCs can give rise to colony-forming units-fibroblasts (CFU-fs), but also the non-adherent fraction (‘Pour-off’ NA-BMCs), indicating the presence of ‘non-adherent’ mesenchymal stem cells (NA-MSCs) in rat bone marrow [16]. First of all, in this study, we examined the clonogenic ability of mouse BMCs using the ‘Pour-off’ method. In agreement with the results obtained from the rat cells, our results showed that both total mouse BMCs and NA-BMCs can give rise to CFU-fs. Also the efficiency of CFU-fs in PO2, PO3, PO4, and PO5 increased 14%, 84%, 161% and 172%, respectively, following the treatment of cells with 1, 25-dihydroxyvitamin D_3_ (10^−8^ M) in mouse BMCs (Figures 2A and B). These results suggest that using of the “Pour-off” method on cultures can maximize the quantity of BM-MSCs, which can be used as seeding cells for tissue transplantations, from limited numbers of adult mouse BMCs. Pooled BM-MSCs from the primary total and PO1–5 BMC cultures were subcultured for 5 passages. The β-gal transgenic mouse-derived BM-MSCs from both the 1st pour off cultures and the 5^th^ passaged cultures, which were positive for β-gal (Figures 2C) as demonstrated by LacZ cytochemical staining, were used as donor cells for the transplantation.

To compare potential differences between the 1^st^ and passaged BM-MSCs, the β-gal transgenic mouse-derived BM-MSCs from the 1^st^ pour-off cultures (the 1^st^ BM-MSC) and the 5^th^ passaged cultures (the 5^th^ BM-MSC) were further characterized by comparison of expression levels of markers of pluripotent stem cells, matrix metalloproteinases (MMPs), protein expression profile, and differentiation potential into cardiomyocytes or vascular endothelial cells. Our results showed that mRNA levels of markers for pluripotent stem cells (OCT-4, CXCR4, LIF-R, Dppa3, Nanog, MET-R, Rex-1 and RIF-1) (Figures 2D–K), for cardiac-committed stem cells (Csx and GATA-4) (Figures 2L, M) or for vascular endothelial-committed stem cells (VE-Cadherin) (Figures 2N) in the 1^st^ BM-MSCs were significantly higher than those in the 5^th^ passage BM-MSCs. These results indicate that the stemness maintenance of the 1^st^ BM-MSCs is superior to that of the 5^th^ BM-MSCs. Our results also showed that mRNA levels of MT1-MMP, MMP-2 and MMP-9 (Figures 2O–Q) in the 1^st^ BM-MSCs were higher than those in the 5^th^ passage BM-MSCs. This implies that migration properties of the 1^st^ BM-MSCs may be superior to that of the 5^th^ BM-MSCs.

**Figure 3:**
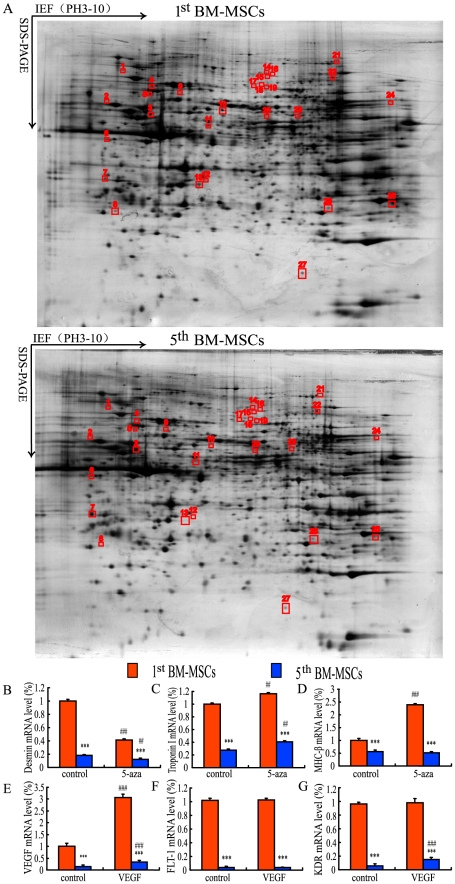
Protein expression profile and differentiation of the primary and 5^th^ passaged BM-MSCs toward cardiocytes and vascular endothelial cells *in vitro*. (A) The representative images of silver stained 2-DE gel of the proteins from the primary pour-off cultures (upper panel, 1^st^ BM-MSCs) and the 5^th^ passaged cultures (low panel, 5^th^ BM-MSCs). (B) Desmin, (C) Troponin I, (D) MHC-ß, (E) VEGF, (F) FLT-1 and (G) KDR mRNA levels were examined in BM-MSCs from the primary pour-off cultures (1^st^ BM-MSCs) and the 5^th^ passaged cultures (5^th^ BM-MSCs) induced by 5′-azacytidine (B–D) and VEGF (E–G) by real-time RT-PCR. The mRNA levels were calculated as a ratio to the GAPDH mRNA level and expressed relative to levels of the 1^st^ BM-MSCs. Each value was the means±S.E.M. of 5 determinations. **: P<0.01; ***: P<0.001 compared with the 1^st^ BM-MSCs.

The comparison of protein expression profile was performed by 2-D electrophoresis (Figures 3A) and mass spectrometric analysis. Our results showed that 27 protein spots were identified to be differentially expressed by the 1^st^ BM-MSCs for at least 5 times in comparison to the 5^th^ BM-MSCs. Among those proteins, 24 proteins were down-regulated and 3 proteins were up-regulated in the 5^th^ BM-MSCs. Some proteins among them have been known to be involved in regulating cellular functions of stem cells, which include adhesion, homing, engraftment, angiogenesis, anti-apoptosis, protecting cells from oxidative stress, and modulating inflammatory and immune responses (Table S1).

To compare differentiation potential into cardiomyocytes or vascular endothelial cells between the 1^st^ BM-MSCs and the 5^th^ BM-MSCs, 5′-aza and VEGF were used for differentiation of BM-MSC into cardiomyocytes and vascular endothelial cells, respectively, and gene expressions of cardiomyocyte markers or vascular endothelial cell markers were examined by real-time RT-PCR. Results showed that mRNA levels of either immature cardiomyocyte marker, desmin or mature cardiomyocyte markers, troponin I and MHC-β in the 1^st^ BM-MSCs were higher than those in the 5^th^ BM-MSCs in the absence or presence of 5′-azacytidine. Also following the treatment of 5′-azacytidine, mRNA levels of desmin were downregulated while mRNA levels of troponin I were upregulated either in the 1^st^ BM-MSCs or in the 5^th^ BM-MSCs, and mRNA levels of MHC-β was upregulated in the 1^st^ BM-MSCs while they remained unchanged in the 5^th^ passage BM-MSCs (Figures 3B–D). mRNA levels of either VEGF or its receptors, KDR and FLT-1, in the 1^st^ BM-MSCs were higher than those in the 5^th^ BM-MSCs in the absence or presence of VEGF. Following the treatment of VEGF, mRNA levels of VEGF were upregulated, while mRNA levels of FLT-1 either in the 1^st^ BM-MSCs or in the 5^th^ BM-MSCs remained unchanged. mRNA levels of KDR remained unchanged in the 1^st^ BM-MSCs while they were upregulated in the 5^th^ BM-MSCs (Figures 3E–G). These results suggest that the 1^st^ BM-MSCs have higher differentiation potential into cardiomyocytes or vascular endothelial cells than the 5^th^ BM-MSCs do.

### Distribution of donor cells in MI areas and infarct border zones following the transplantation

**Figure 4:**
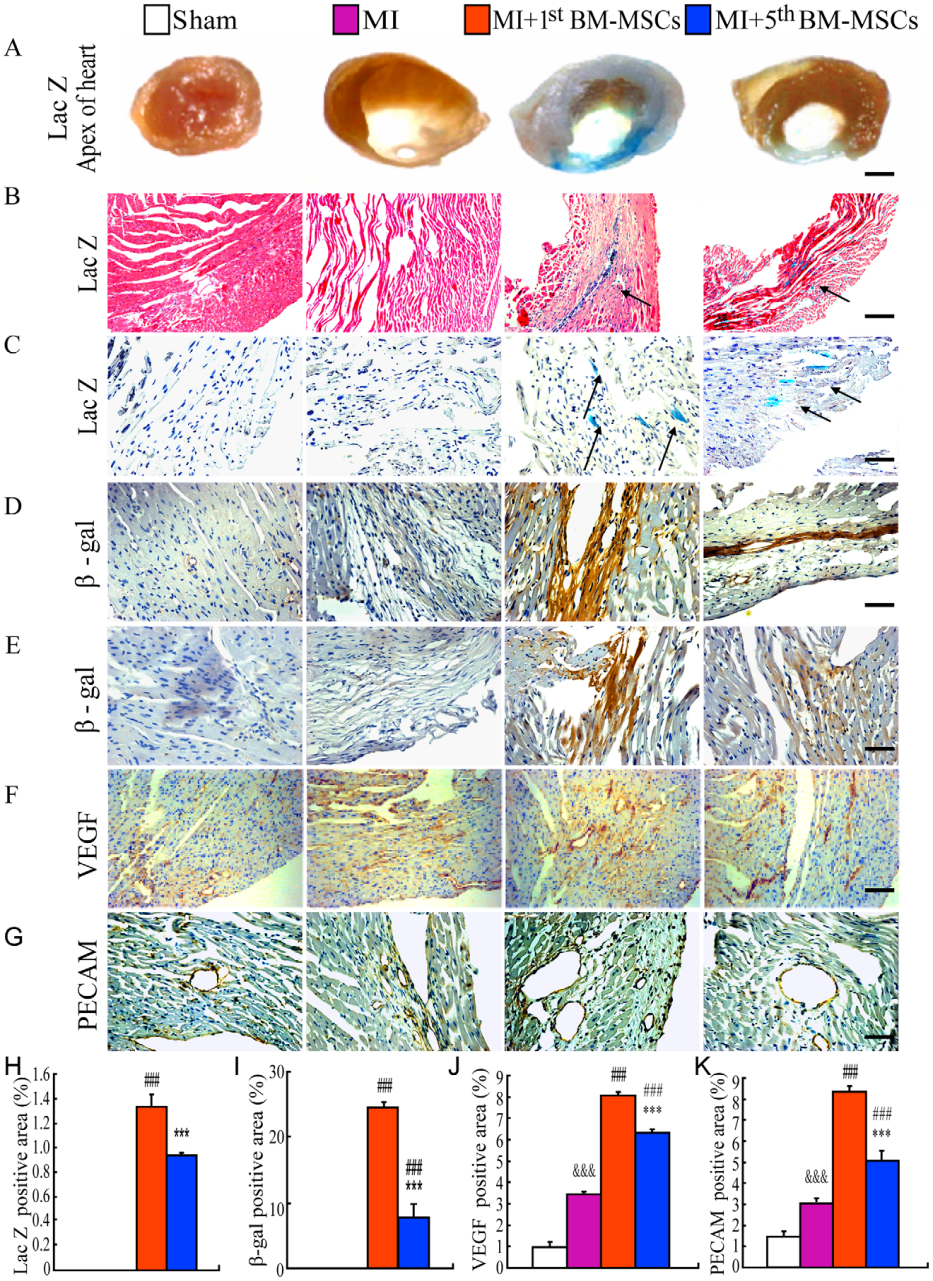
Distribution of donor cells in MI areas and infarct border zones following the transplantation. (A) Representative graphs of left ventricles from sham, MI, MI with the transplantation with the 1^st^ BM-MSCs (MI+1^st^ BM-MSCs) or with the 5^th^ BM-MSCs (MI+5^th^ BM-MSCs) stained histochemically for ß-Gal activity. Representative micrographs of (B) MI areas stained histochemically for ß-Gal activity and counterstained with H & E and (C) infarct border zone stained histochemically for ß-Gal activity and counterstained with Hematoxylin. Representative micrographs of (D) MI areas and (E) infarct border zone immunostained for ß-Gal and counterstained with Hematoxylin. (F) Representative micrographs of infarct border zone immunostained for VEGF. (G) Representative micrographs of infarct border zone immunostained for PECAM. Scale bars in A–G represent 4000, 50, 25, 25, 25, 50 and 25 µm, respectively. (H) LacZ positive areas, (I) ß-Gal immunopositive areas, (J) VEGF immunopositive areas and (K) PECAM immunopositive areas were quantitated by computer-assist image analysis and presented as Means±S.E.M. of determinations in five animals of each group. ***, P<0.001 compared with MI+1^st^ BM-MSCs group. ###, P<0.001 compared with MI group. &&&, P<0.001 compared with sham group.

To trace the β-gal^+^ primary and the 5^th^ BM-MSCs in recipient hearts, we analyzed heart specimens after 6 weeks, following the transplantation using LacZ histochemical staining and immunohistochemical staining for β-gal. Our results showed that no LacZ^+^ staining was detected in the heart from either sham or MI mice without the transplantation, whereas patch-like LacZ^+^ staining was detected at MI areas from both the MI+1st BM-MSCs group and the MI+5^th^ BM-MSCs group, but the area of LacZ^+^ staining in the hearts from the MI+1^st^ BM-MSCs group was greater than that from the MI+5^th^ BM-MSCs group (Figures 4A). Moreover, as revealed by H.E. counter-staining, diffusive LacZ^+^ cells were only seen in the hearts from either the MI+1^st^ BM-MSCs group or the MI+5^th^ BM-MSCs group, and numbers of LacZ^+^ cells in the hearts from the MI+1^st^ BM-MSCs group were greater than those from the MI+5^th^ BM-MSCs group (Figures 4B and C). In agreement with the LacZ staining results, immuohistochemical staining revealed that β-gal^+^ cells were only seen in the hearts from either the MI+1^st^ BM-MSCs group or the MI+5^th^ BM-MSCs group, and that numbers of β-gal^+^ cells in the hearts from the MI+1^st^ BM-MSCs group were greater than those from the MI+5^th^ BM-MSCs group (Figures 4D and E). Furthermore, as shown by histomorphometric analysis, area of cells either positive for LacZ or β-gal in the hearts from the MI+1^st^ BM-MSCs group was greater than that from the MI+5^th^ BM-MSCs group, and notably the area of cells positive for β-gal was greater than that of cells positive for LacZ (Figures 4F and G), indicating that not all of the cells positive for β-gal had β-galactosidase activity. These results suggested that properties of cell survival and homing into MI area for the 1^st^ BM-MSCs are superior to those for the 5^th^ BM-MSCs.

### Differentiation capacity of donor cells towards cardiomyocytes or neovasculature and vascular endothelial cells in infarct border zones following the transplantation

**Figure 5:**
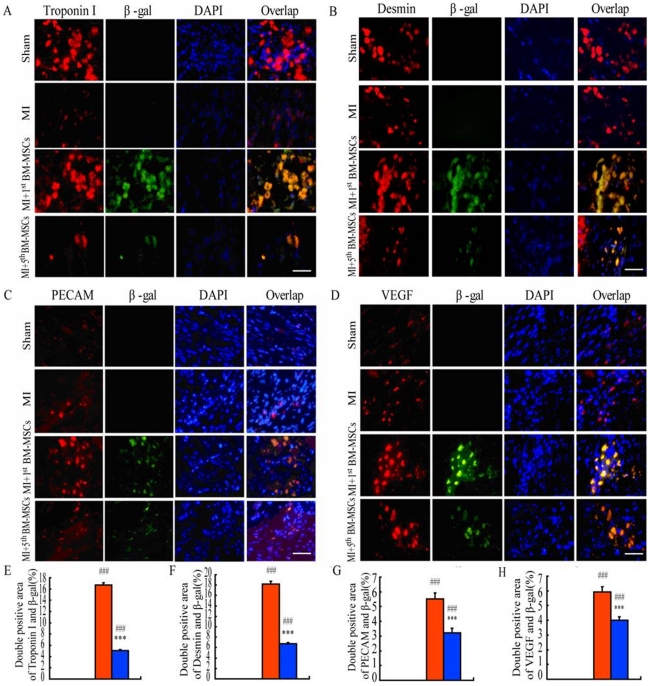
Differentiation capacity of donor cells towards cardiomyocytes or neovasculature and vascular endothelial cells in infarct border zones following the transplantation. Representative micrographs of infarct border zones from sham, MI, MI with the transplantation with the 1^st^ BM-MSCs (MI+1^st^ BM-MSCs) or with the 5^th^ BM-MSCs (MI+5^th^ BM-MSCs) stained immunofluresence for troponin I (red, left panel) and ß-gal (green, 2^nd^ panel), with DAPI for nuclei (blue, 3^rd^ panel) and overlap (4^th^ panel). (B) Representative micrographs of infarct border zones stained immunofluresence for desmin (red, left panel) and ß-gal (green, 2^nd^ panel), with DAPI for nuclei (blue, 3^rd^ panel) and overlap (4^th^ panel). (C) Representative micrographs of infarct border zones stained immunofluresence for PECAM (red, left panel) and ß-gal (green, 2^nd^ panel), with DAPI for nuclei (blue, 3^rd^ panel) and overlap (4^th^ panel). (D) Representative micrographs of infarct border zones stained immunofluresence for VEGF (red, left panel) and ß-gal (green, 2^nd^ panel), with DAPI for nuclei (blue, 3^rd^ panel) and overlap (4^th^ panel). (E) Double positive areas for troponin I and ß-Gal, (F) Double positive areas for desmin and ß-Gal, (G) Double positive areas for PECAM and ß-Gal, and (H) Double positive areas for VEGF and ß-Gal were quantitated by computer-assist image analysis and presented as Means±S.E.M. of determinations in five animals of each group. Scale bars in A–D represent 10 µm. ***, P<0.001 compared with MI+1^st^ MSCs group. ###, P<0.001 compared with MI group.

To assess differentiation capacity of donor cells towards cardiomyocytes or neovasculature and vascular endothelial cells in infarct border zones following the transplantation, double immunofluorescence staining for β-gal and troponin I/desmin or for β-gal and PECAM/VEGF were performed after 6 weeks following the transplantation. Our results showed that double positive areas β-gal and troponin I/desmin were only detected in the MI+1^st^ BM-MSCs group or the MI+5^th^ BM-MSCs group, and the percentage of those double positive areas in the MI+1^st^ BM-MSCs group was significantly higher than those in the MI+5^th^ BM-MSCs group (Figures 5A, B, E and F). These results indicate that the differentiation capacity towards cardiomyocytes of the 1^st^ BM-MSCs, as donor seeding cells following MI, is greater than that of the 5^th^ BM-MSCs.

It was also found that areas of vasculature stained positive for PECAM/VEGF in the MI group were greater than those in the sham group. The transplantation of the 1^st^ BM-MSCs or the 5^th^ BM-MSCs into MI models resulted in increased percentages of areas of vasculature stained positive for VEGF/PECAM in infarct border zones. Also, the percentage of those positive areas in the MI+1^st^ BM-MSCs group was significantly greater than those in the MI+5^th^ BM-MSCs group. Double positive areas for β-gal and VEGF/PECAM were only detected in the MI+1^st^ BM-MSCs group or the MI+5^th^ BM-MSCs group. The percentage of those double positive areas in the MI+1^st^ BM-MSCs group was significantly greater than those in the MI+5^th^ BM-MSCs group (Figures 5C, D, G and H). These results indicate that: MI results in neovasculature formation in MI areas and infarct border zones; the transplantation of BM-MSCs can promote neovasculature formation in the affected areas; the transplantation of the 1^st^ BM-MSCs has a greater effect on neovasculature formation than that of the 5^th^ BM-MSCs and differentiation capacity towards vasculature tissue of the 1^st^ BM-MSCs as donor seeding cells following MI is greater than that of the 5^th^ BM-MSCs.

### Assessments of cell proliferation and apoptosis in the myocardium following the transplantation of the 1^st^ or the 5^th^ BM-MSCs into the MI models

**Figure 6:**
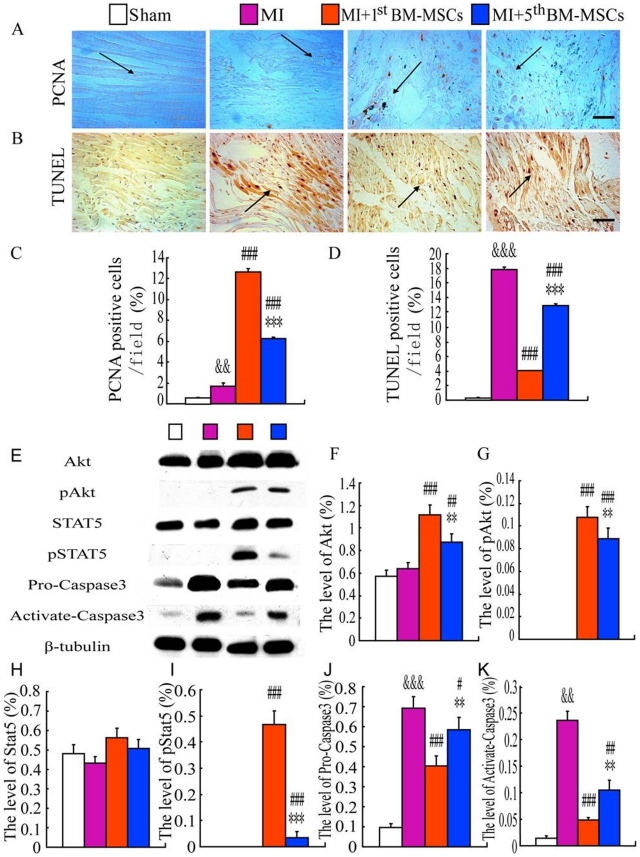
Assessments of cell proliferation and apoptosis in the myocardium following the transplantation of the 1^st^ or the 5^th^ BM-MSCs into the MI models. Representative micrographs of infarct border zones from sham, MI, MI with the transplantation with the 1^st^ BM-MSCs (MI+1^st^ BM-MSCs) or with the 5^th^ BM-MSCs (MI+5^th^ BM-MSCs) (A) stained immunohistochemically for PCNA and (B) with TUNEL. Scale bars in A and B represent 25 µm. (C) PCNA positive cells/field and (D) TUNEL positive cells/field were quantitated by computer-assist image analysis and presented as Means±S.E.M. of determinations in five animals of each group. (E) Western blots of infarct border area extracts were carried out for expression of Akt, p-Akt, STAT5, pSTAT5, Pro-caspase3, Activate-caspase3 and β-tubulin was used as a loading control. (F) Akt, (G) p-Akt, (H) STAT5, (I) pSTAT5, (J) Pro-Caspase3, (K) Activate-caspase3 protein levels relative to β-tubulin protein levels were assessed by densitometric analysis and were presented as Means±S.E.M. of determinations in five animals of each group. **, P<0.01; ***, P<0.001 compared with MI+1^st^ BM-MSCs group; #, P<0.05; ##, P<0.01; ###, P<0.001 compared with MI group; &&& P<0.001 compared with sham group.

To assess cell proliferation and apoptosis in the myocardium, immunohistochemical staining for PCNA and TUNEL assay for apoptotic nuclei in infarct border zones was performed after 6 weeks following the transplantation of the 1^st^ or the 5^th^ BM-MSCs into the MI models. Our results showed that: i) PCNA-positive cells were occasionally present in the myocardium and on the periphery of heart vasculatures in the sham group and were increased in MI areas and infarct border zones in the MI group; the percentage of PCNA-positive cells in infarct border zones either in the MI+1^st^ BM-MSCs group or the MI+5^th^ BM-MSCs group was significantly greater than those in MI group; the percentage of PCNA-positive cells in infarct border zones in the MI+1^st^ BM-MSCs group was significantly greater than that in the MI+5^th^ BM-MSCs group (Figures 6A, and C). TUNEL assay results showed that TUNEL-positive nuclei were occasionally present in the myocardium in the sham group and were increased in infarct border zones in the MI group; the percentage of TUNEL-positive nuclei in infarct border zones either in the MI+1^st^ BM-MSCs group or the MI+5^th^ BM-MSCs group was reduced significantly compared to that in MI group; the percentage of TUNEL-positive nuclei in infarct border zones in the MI+1^st^ BM-MSCs group was reduced significantly compared to that in the MI+5^th^ BM-MSCs group (Figures 6B and D). Those results indicate that MI results in cell proliferation and apoptosis in infarct border zones; the transplantation of BM-MSCs into MI models enhanced cell proliferation and attenuated apoptosis in infarct border zones; the 1^st^ BM-MSCs has a more evident impact on either stimulating cell proliferation or inhibiting cell apoptosis in infarct border zones than the transplantation of the 5^th^ BM-MSCs.

Furthermore, to investigate possible mechanisms underlying attenuated cell apopotsis in infarct border zones following transplantations of BM-MSCs, we examined the expression of Akt, phospho-Akt (pAkt), STAT5, phospho-STAT5 (pSTAT5), pro-caspase 3 and active caspase 3 in lysate samples prepared from infarct border zones, following the transplantations using Western blotting. Our results showed that: there is no apparent difference in levels of either Akt or STAT5 in lysate samples between the sham group and the MI group and either pAkt or pSTAT5 was undetectable in the two groups; levels of both pro-caspase 3 and active caspase 3 were upregulated in lysate samples from the MI group; interestingly, levels of either Akt or pAkt was upregulated in lysate samples from both the MI+1^st^ BM-MSCs group and the MI+5^th^ BM-MSCs group; pSTAT5 became detectable while STAT5 levels remained unchanged in both the MI+1^st^ BM-MSCs group and the MI+5^th^ BM-MSCs group; Levels of both pro-caspase 3 and active caspase 3 were downregulated in both the MI+1^st^ BM-MSCs group and the MI+5^th^ BM-MSCs group; levels of Akt, pAkt, or pSTAT5 in lysate samples from the MI+1^st^ BM-MSCs group were significantly higher than those in the MI+5^th^ BM-MSCs group and levels of both pro-caspase 3 and active caspase 3 were in lysate samples from the MI+1^st^ BM-MSCs group were significantly lower than those in the MI+5^th^ BM-MSCs group (Figures 6E–K).

### Assessment of cardiac functions following the transplantation of the 1^st^ or the 5^th^ BM-MSCs into MI models

**Figure 7:**
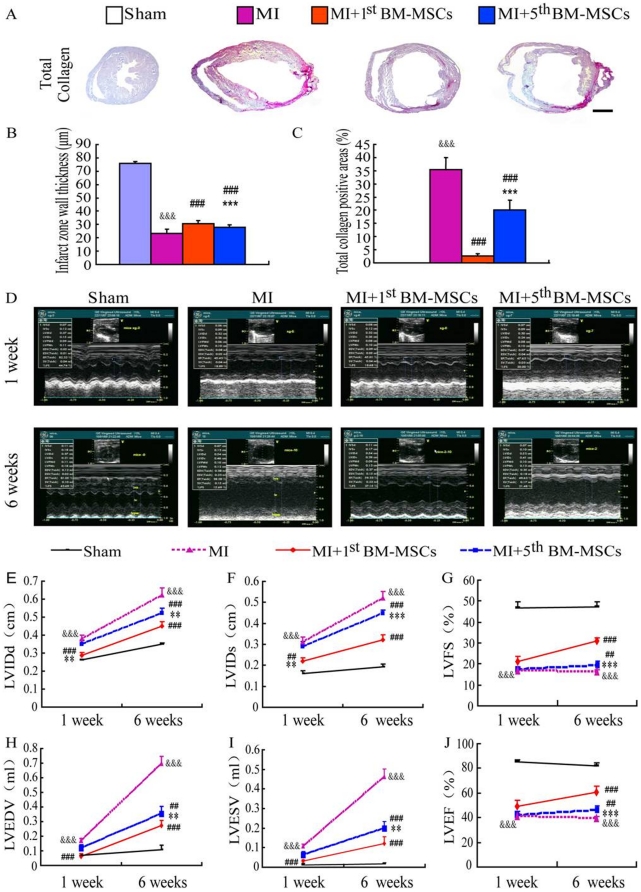
Assessment cardiac functions following the transplantation of the 1^st^ or the 5^th^ BM-MSCs into MI models. (A) Representative graphs of ventricles from sham, MI, MI with the transplantation with the 1^st^ BM-MSCs (MI+1^st^ BM-MSCs) or with the 5^th^ BM-MSCs (MI+5^th^ BM-MSCs) stained histochemically for total collagen. Scale bar represent 800 µm. (B) Infarct wall thickness and (C) total collagen positive areas were quantitated by computer-assist image analysis and presented as Means±S.E.M. of determinations in five animals of each group. (D) Representative echocardiographs recorded after 1 and 6 weeks following the transplantation. (E) LVIDd, (F) LVIDs, (G) LVFS, (H) LVEDV, (I) LVESV and (J) LVEF. Each value is the means ± SEM of determinations in 5 animals of each group. **, P<0.01; ***, P<0.001 compared with MI+1^st^ BM-MSCs group. ##, P<0.01; ###, P<0.001 compared with MI group. &&&, P<0.001 compared with sham group.

By histochemical staining for total collagen and histomorphometric analysis at the 6th week after MI revealed that the percentage of positive areas for total collagen in the left ventricle wall in the MI group was greater than that in the sham group, the transplantation of BM-MSCs into MI models resulted in an increased thickness of the infarct border zone ventricular wall and decreased positive areas for total collagen. Also the thickness of the infarct border zone ventricular wall in the MI+1^th^ BM-MSCs group increased more significantly and positive areas for total collagen decreased more significantly in comparison to the MI+5^st^ BM-MSCs group (Figures 7A–C).

To assess cardiac functions, echocardiographic analyses were performed after 1 or 6 weeks following the transplantation. LVIDd, LVIDs, LVEDV and LVESV were increased significantly while LVFS and LVEF were decreased significantly in the MI group compared to the sham controls. After 1 week following the transplantation, LVIDd, LVIDs, LVEDV and LVESV were decreased while LVFS and LVEF remained unchanged in the MI+1^st^ BM-MSCs compared to the MI group, in contrast, these parameters were not altered in the MI+5^th^ BM-MSCs group. After 6 weeks following the transplantation, LVIDd, LVIDs, LVEDV and LVESV were decreased, whereas LVFS and LVEF were increased in both the MI+1^st^ BM-MSCs and the MI+5^th^ BM-MSCs groups compared to the MI group. The alterations of these parameters were more dramatic in the MI+1^st^ BM-MSCs group compared to the MI+5^th^ BM-MSCs group (Figures 7D–J). These results indicate that the transplantation of either the 1^st^ BM-MSCs or the 5^th^ BM-MSCs can increase the thickness of the infarct border zone ventricular wall, alleviate myocardial fibrosis and improve left ventricular functions. Also, the transplantation of the 1^st^ BM-MSCs has a better therapeutic effect as a means for MI than the transplantation of the repeated passage BM-MSCs.

## Discussion

The transplantation of stem cells is believed to be a promising approach to treatment of ischemic heart diseases, which cannot be cured by medication or surgical interventions. BM-MSCs are highly self-renewable multipotent stem cells and have been widely used in therapeutic transplantation and tissue engineering [2]. However, BM-MSCs are not immortal and their capacities of proliferation and differentiation are known to be impaired through the repeated passaging process *in vitro* [26]. Repeated passaging, a conventional approach to amplify BM-MSCs for sufficient numbers required for a successful transplantation, is known to compromise the stem cell biological characteristics of BM-MSCs [16]. Unsatisfactory therapeutic effect is likely to be attributable to the utilization of repeated passaging BM-MSCs, which only have an impaired regeneration capacity for either cardiac muscles or vascular tissues, as seeding cells for heart transplantation following acute MI. Therefore it is imperative to seek a population of BM-MSCs which have fine stem cell biological characteristics, and can be amplified extensively, for satisfactory therapeutic effect following acute MI. BM-MSCs are generally regarded as a highly adhesive fibroblast-like cell type. However, our previous results [16], in agreement with other reports [17], have indicated the existence of a population of non-adherent and round cells with self-renewal and differentiation potency towards multiple tissues, and these cells are also capable of forming CFU-Fs *in vitro*. Accordingly, we have developed the “Pour-off” method to amplify primary BM-MSC and have demonstrated that the treatment of the cells with 1,25-(OH)_2_D_3_ enhances the formation of CFU-Fs. In this study, we prepared primary BM-MSCs as seeding cells for the transplantation using the “Pour-off” culture method. In comparison to the 5^th^ passage BM-MSCs, the 1^st^ BM-MSCs had better therapeutic effects in the mouse MI model in that they were readily being engrafted into the scarred myocardium and border zone, proliferating gradually *in situ*, forming mature vessels and cardiomocytes, promoting angiopoiesis, reversing wall thinning in scarred areas and improving cardiac functions. Those results indicated that transplantation of the 1^st^ BM-MSCs prepared using the “Pour-off” culture method has a better therapeutic effect on acute MI than that of repeated passage BM-MSCs.

β-galactosidase (β-gal) encoded by the bacterial gene *lacZ* is a very effective molecular marker to trace the migration, distribution, proliferation, and differentiation of donor cells *in vivo* for their roles in tissue repair following injury. In this study, we used BM-MSCs derived from β-galactosidase transgenic mice as donor cells for intramyocardial transplantation in male C57BL/6J wild type mice following acute MI. We also performed histochemical staining for LacZ and immunohistochemical staining for β-gal to show the migration/distribution and differentiation of the donor cells. Our results indicated that at the 6^th^ week following the immediate local transplantation of β-gal^+^ BM-MSCs, β-gal^+^ cells were seen at infarct foci, and some of the cells expressed markers of mature cardiomyocytes. It has been shown that the peak of inflammatory phase occurs at days 1∼3 [27], and survival of transplanted BM-MSC was likely to be compromised if the cells were transplanted during this period. It is known that muscular vasculatures are formed at ∼20 days following MI [27]. The migration of transplanted BM-MSC was likely to be compromised if the cells were transplanted during this stage. Moreover levels of some growth factors such as VEGF reach a peak at day 7 following MI [27], so the best time points for the transplantation of BM-MSC are likely to be between day 7 and day 14 following MI to reverse left ventricular remodeling. Although certain therapeutic effects were demonstrated in this study by immediate transplantation of BM-BMCs, following acute MI, whether transplantation of BM-MSCs between day 7 and day 14 following MI has a better therapeutic effect remains to be examined.

Recently, the existence of a population of small embryonic-like CXCR4-SSEA-Oct4^+^ stem cells expressing markers for embryonic stem cells and tissue-committed progenitor cells in adult bone marrow has been demonstrated [28]. The CXCR4-SSEA-Oct4^+^ stem cells are self-renewal proliferating cells and play an active role in tissue repair following MI [29]. This suggests the existence of very small embryonic-like stem cells (VSELs) in adult bone marrow. However, the scarcity of CXCR4-SSEA-Oct4^+^ stem cells and the lack of an effective amplification method put a limit on its clinical application. In this study, we found that BM-MSCs *in vitro* express markers for embryonic stem cells, cardiac-committed stem cells, or vascular endothelial-committed stem cells. Gene expression levels of the stem cell markers by the 1^st^ BM-MSCs are significantly higher than those expressed by the 5^th^ passage BM-MSCs, and expressions of some of those markers by the 1^st^ BM-MSCs are higher than those by total BMCs. This implies amplification of *VSELs* in the process of amplifying the 1^st^ BM-MSCs and differentiation potency towards cardiomyocytes and vascular endothelial cells. Interestingly Oct4, a key transcription factor in the induction of somatic cell towards pluripotent stem cells, is expressed at higher levels in the 1^st^ BM-MSCs, in comparison to the 5^th^ passage BM-MSCs. PKM2 is known to upregulate transcription levels of Oct4 [30]. Our results from MS analysis following 2-DE indicated that the 1^st^ BM-MSCs express PKM2 at a higher level in comparison to the 5^th^ passage BM-MSCs. This could be the reason why the 1^st^ BM-MSCs express higher levels of Oct4. Furthermore, levels of either Csx or GATA-4 expressed by the 1^st^ BM-MSCs were higher than those by the 5^th^ passage BM-MSCs. Likewise, in this study, our results indicate that the differentiation capacities of the 1^st^ BM-MSCs towards either cardiomyocytes or vascular endothelial cells is superior to those of the 5^th^ passage BM-MSCs *in vivo* and *in vitro*. Undoubtedly stem cell biological characteristics of donor/seeding cells is a key factor for improving therapeutic effect of BM-MSCs transplantation following acute MI.

Recent studies suggest that the transplantation of BM-MSCs may not only directly induce neovasculature formation but also enhance indirectly neovasculature formation via autocrine factors such as VEGF and cytokines [31], [32]. It has been shown that the transplantation of BM-MSCs following MI results in the elevation of VEGF levels for 2 months [11]. In agreement with those studies, our results indicate that the transplantation of BM-MSCs enhances neovasculature formation in MI areas and infarct border zones. More interestingly, our results from MS analysis following 2-D electrophoresis indicated that the 1^st^ BM-MSCs express higher levels of annexin A3, annexin A2, or hn RNP K in comparison to the 5^th^ passage BM-MSCs. Those three proteins are known to enhance neovasculature formation via paracrine mechanisms. Annexin A3, a newly discovered pro-angiogenic factor, can induce VEGF via the HIF pathway [33]. Annexin A2 can regulate neoangiogenesis *in vivo* [34], and hn RNP K can upregulate VEGF mRNA level via stimulating angiotensin II. Additionally it has been shown that newly regenerated cardiomyocytes are capable of enhancing neovasculature formation via secreting VEGF [35]. In this study, our results indicate that the 1^st^ BM-MSCs have greater differentiation potency towards cardiomyocytes in comparison to the 5^th^ BM-MSCs, therefore it is likely that the 1^st^ BM-MSCs have more profound pro-angiogenic effect than the 5^th^ BM-MSCs do. Differences between the 1^st^ BM-MSCs and the 5^th^ BM-MSCs in the two aspects mentioned above may explain why more VEGF positive reactions are present in the infarct areas and border zones in the MI+1^st^ BM-MSCs group than those in the MI+5^th^ BM-MSCs group.

SDF-1/CXCR4 axis seems particularly important for the homing, chemotaxis, engraftment, and retention of stem/muscle progenitor cells within ischemic myocardium. SDF-1 released from the ischemic myocardium improves cardiac performance through its effects on pro-angiogenesis, cardio-protective, anti-apoptotic, and wound healing [36]. Moreover, SDF-1 is a potent chemoattractant for cells expressing CXCR4 [3]. It is evident that bone marrow and skeletal muscles contain a small population of cells positive for CXCR4 antigen and expressing genes specific for early muscle-committed stem/progenitor cells [3]. In this study, we found that the 1^st^ BM-MSCs expressed CXCR4 at obviously higher levels in comparison to the 5^th^ passage BM-MSCs, suggesting that primary BM-MSCs might have more motility in response to chemoattractants such as SDF-1. Previous studies have shown that the overexpression of CXCR4 in BM-MSCs has been correlated to upregulated gene expressions of MMPs, such as MT1-MMP and MMP-9, under hypoxic conditions [37]. MMPs are known to play an important role in cell migrations. In this study, we found that mRNA levels of MMP-2, MMP-9 and MT1-MMP in the 1^st^ BM-MSCs are higher than those in the 5^th^ BM-MSCs, and this may be attributable to high levels of CXCR4 levels in the primary BM-MSCs. Meanwhile, our results from MS analysis following 2-DE indicated that the 1^st^ BM-MSCs express higher levels of TRAP1, which is known to induce MMP-9 expression in cardiomyocytes [38], in comparison to the 5^th^ passage BM-MSCs. Previous studies suggest that high levels of MMPs may contribute to softening the collagenous areas, enhancing the ability of CXCR4^+^-MSCs to cross the reconstituted basement membrane for the generation of new myocytes [37]. Additionally annexin A2, which is highly expressed in the 1^st^ BM-MSCs and is known to have an effect on fibrinolysis [34], may have a role in the reorganization of collagen fibril in the MI areas and border zones. Furthermore, annexin A2 together with Lgals, another protein highly expressed in the 1^st^ BM-MSCs, may both regulate the adhesion, homing, migration, and engraftment of BM-MSCs following the transplantation.

In this study, our data of immunohistochemical staining for PCNA showed that a number of PCNA-positive cells in MI areas and infarct border zones in the MI+1^st^ BM-MSCs group was significantly greater than that in the MI+5^th^ BM-MSCs group in MI areas and border zones. This indicates a strong amplification potential of the primary BM-MSCs *in vivo*. Relevantly our data of MS analysis following 2-DE, showed mcm7, a cell proliferation-promoting protein [39], is highly expressed by the 1^st^ BM-MSCs. In contrast, the isoform 2 of src kinase-associated phosphoprotein, a cell proliferation-inhibiting protein [40], is upregulated in the 5^th^ passage BM-MSCs.

In this study, we found that, in comparison to the transplantation of the 5^th^ BM-MSCs, the transplantation of the 1^st^ BM-MSCs has more evident anti-apopotsis effect in MI areas and border zones. Multiple mechanisms may underlie this difference. First of all, as shown by MS analysis following 2-DE, lgals, a protein known to have anti-apoptotic effect through enhancing p-AKT levels [41], is highly expressed in the primary BM-MSCs. p-Akt may contribute to the protection of ischemic myocardium, possibly through enhanced expression of VEGF, as well as by decreasing ROS [10]. Secondly, as identified by MS analysis following 2-DE, trap1, a protein known to have an important role in anti-apoptosis through suppressing the formation of ROS [41], is highly expressed in the 1^st^ BM-MSCs. Meanwhile, as identified by MS analysis following 2-DE, catalase and stip1 are highly expressed in the 1^st^ BM-MSCs, and these two proteins may rescues cells from apoptosis by protecting cells against oxidative stress by H_2_O_2_ [42], [43]. Furthermore, efhd2, a protein known to contribute to BCR-induced apoptosis by enhancing BCR signal [44], is highly expressed in the 5^th^ BM-MSCs. Ultimately, as shown by our immunoblotting results, levels of either total caspase3 or its active form, which play a critical role in the execution of apoptosis, is down-regulated in the 1^st^ BM-MSCs transplanted heart more obviously than the 5^th^ BM-MSCs transplanted heart. This implies anti-apoptotic property of the 1^st^ BM-MSCs is superior to that of the 5^th^ BM-MSCs. Our TUNEL assay results support this possibility. Additionally, p-STAT5 level in lysate samples prepared from primary BM-MSCs transplanted hearts was higher than that in those prepared from the 5^th^ BM-MSCs transplanted hearts, raising the possibility that the activation of the STAT5 pathway may inhibit caspase-3-mediated apoptosis in the primary BM-MSCs. To sum up, in comparison to the transplantation of the 5th passage BM-MSCs, the transplantation of the 1^st^ BM-MSCs more effectively leads to the efficient activation of Akt/STAT5 pathway. This inhibits caspase-3-mediated apoptosis and efficient inhibition of ROS formation, which protects cardiomyocytes from ischemic injury resulted from oxidative stress. It may also play a better role in preventing post-MI ventricular remodeling, through assisting viable ventricular cardiomyocytes to escape from the immune reaction induced by BCR, and /or alleviating inflammation in the MI areas and border zones.

In summary, in the present study, a novel “Pour-off” method was used to obtain sufficient numbers of 1^st^ BM-MSCs with a good stem cell quality. Following intramyocardial transplantion into the mouse MI model, in comparison to the 5^th^ BM-MSCs, the 1^st^ BM-MSCs were more capable of rapidly migrating, proliferating *in situ*, and differentiating into mature vascular endothelial cells and cardiomyocytes. Meanwhile, undifferentiated 1^st^ BM-MSCs may contribute to the infarct-healing process and to improving post-MI cardiac performance through its paracrine effects on promoting cell migration, homing, and proliferation and neovasculature formation, and preventing apoptosis in the MI areas and border zones. Therefore, using the 1^st^ BM-MSCs obtained using the “Pour-off” method as seeding cells for intramyocardial transplantation following acute MI in mice has a better therapeutic effect than using the 5^th^ BM-MSCs obtained from conventional repeated passaging.

## Supporting Information

Table S1 Summary of the differential proteins.
(DOC)
