# Correction: An Improved Transplantation Strategy for Mouse Mesenchymal Stem Cells in an Acute Myocardial Infarction Model

**DOI:** 10.1371/journal.pone.0316592

**Published:** 2024-12-26

**Authors:** Jianliang Jin, Yingming Zhao, Xiao Tan, Chun Guo, Zhijian Yang, Dengshun Miao

In Fig 4, there is an error in panel E. The image of the β-galactosidase immunohistochemistry for the MI+1st BM-MSCs group is incorrect. Please see the correct Fig 4 here.

**Fig 4:**
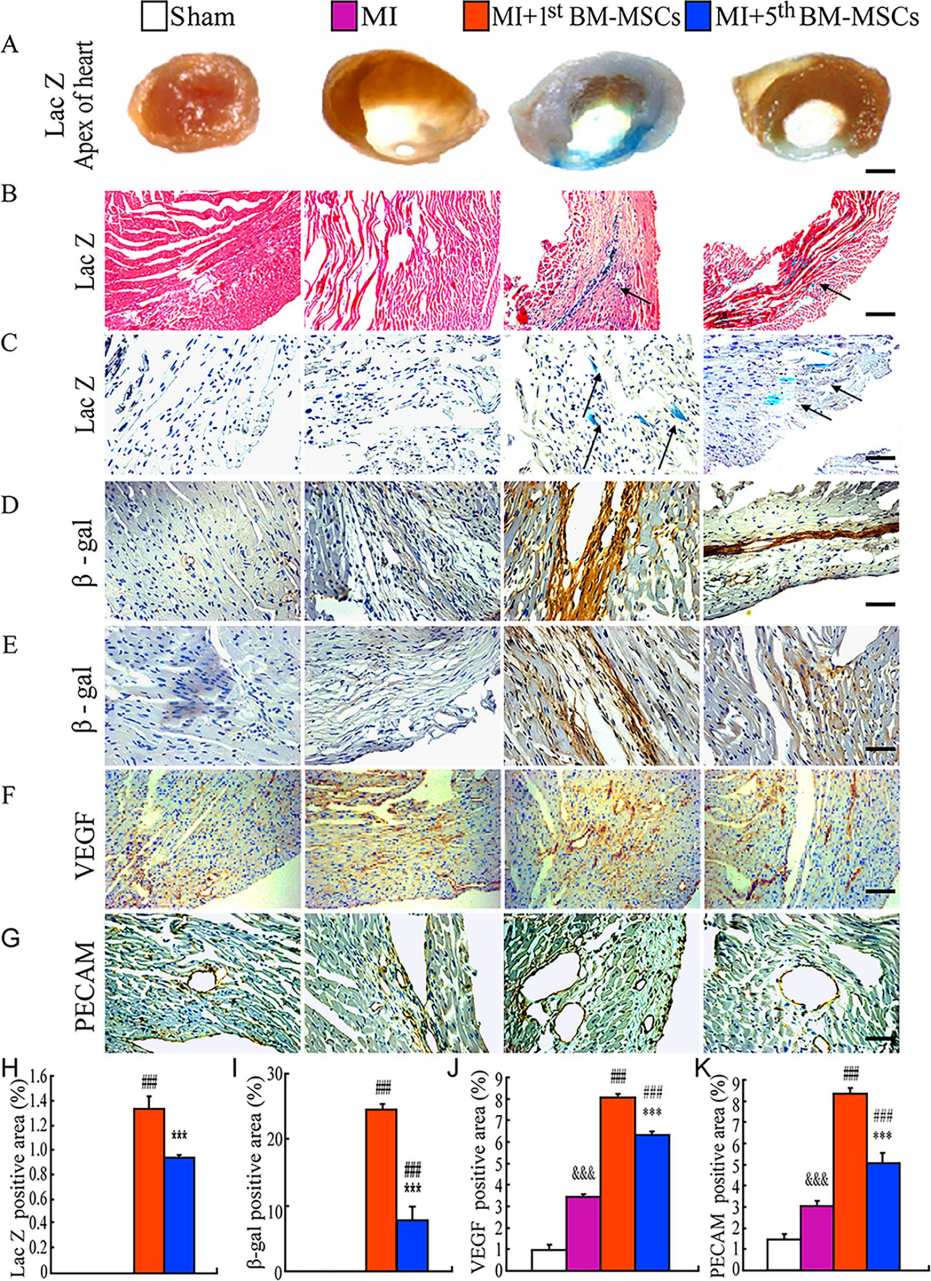
Distribution of donor cells in MI areas and infarct border zones following the transplantation. (A) Representative graphs of left ventricles from sham, MI, MI with the transplantation with the 1st BM-MSCs (MI+1st BM-MSCs) or with the 5th BM-MSCs (MI+5th BM-MSCs) stained histochemically for ß-Gal activity. Representative micrographs of (B) MI areas stained histochemically for ß-Gal activity and counterstained with H & E and (C) infarct border zone stained histochemically for ß-Gal activity and counterstained with Hematoxylin. Representative micrographs of (D) MI areas and (E) infarct border zone immunostained for ß-Gal and counterstained with Hematoxylin. (F) Representative micrographs of infarct border zone immunostained for VEGF. (G) Representative micrographs of infarct border zone immunostained for PECAM. Scale bars in A–G represent 4000, 50, 25, 25, 25, 50 and 25 µm, respectively. (H) LacZ positive areas, (I) ß-Gal immunopositive areas, (J) VEGF immunopositive areas and (K) PECAM immunopositive areas were quantitated by computer-assist image analysis and presented as Means±S.E.M. of determinations in five animals of each group. ***, P<0.001 compared with MI+1st BM-MSCs group. ###, P<0.001 compared with MI group. &&&, P<0.001 compared with sham group.
