# Supplementary material for: An Improved Transplantation Strategy for Mouse Mesenchymal Stem Cells in an Acute Myocardial Infarction Model
Source: PLoS One. 2011 Jun 17;6(6):e21005. doi: 10.1371/journal.pone.0021005 (PMC3117862; doi:10.1371/journal.pone.0021005)
Supplement: Table S1 — Summary of the differential proteins. (DOC) [file pone.0021005.s001.doc]

Table 2： Summary of the differential proteins

| Spot  NO. | Serial  Number | Sequence  Coverage | Score | Down  /UP | Calculated PI  value/Nominal  mass | Protein description | Function |
| --- | --- | --- | --- | --- | --- | --- | --- |
| 1  2  3  4  5  6  7  8  9  10  11  12  13  14  15  16  17  18  19  20  21  22  23  24  25  26  27 | IPI00331556  IPI00830879  IPI00869393  IPI00817026  IPI00230394  IPI00310091  IPI00227299  IPI00226872  IPI00136703  IPI00890005  IPI00119113  IPI00387515  IPI00132722  IPI00652436  IPI00126396  IPI00330862  IPI00114375  IPI00121514  IPI00132762  IPI00331182  IPI00137409  IPI00330523  IPI00752578  IPI00109354  IPI00407130  IPI00890027  IPI00468203  IPI00881629 | 21%  29%  25%  20%  40%  38%  56%  34%  17%  30%  48%  31%  42%  35%  29%  26%  52%  35%  25%  22%  34%  25%  27%  30%  25%  46%  33%  49% | 103  97  76  70  134  173  188  71  69  74  218  111  170  152  125  83  308  150  108  142  235  112  92  64  88  109  73  101 | Up  Up  Up  Down  Up  Up  Up  Down  Up  Up  Up  Up  Up  Up  Up  Down  Up  Up  Up  Up  Up  Up  Up  Up  Up  Up  Up  Up | 5.15/94872  4.71/47993  7.72/60043  4.57/40267  5.11/66973  5.00/66079  5.06/53712  5.07/26784  5.40/42971  5.69/48708  5.57/56857  5.98/44875  5.33/36520  5.23/42053  5.98/81900  5.83/69478  5.95/62638  6.40/63170  6.25/80501  6.01/83347  7.23/68272  6.83/80498  6.82/64647  8.03/58779  7.18/58378  9.51/19376  7.55/38937  6.48/16298 | Hspa4 Heat shock 70 kDa protein 4  Zfp259 48 kDa protein  Cat catalase  Isoform 2 of Src kinase-associated phosphoprotein 2  Lmnb1 Lamin-B1  Ppp2r1a Serine/threonine-protein phosphatase 2A 65 kDa regulatory subunit  Vim Vimentin  Efhd2 16 days embryo head cDNA,  RIKEN full-length enriched library, clone  Ckb Creatine kinase B-type  Hnrnpk Isoform 3 of Heterogeneous nuclear ribonucleoprotein K  Atp6v1b2 Vacuolar ATP synthase subunit B,brain isoform  Lpxn Leupaxin  Anxa3 Annexin A3  Actg1 In vitro fertilized eggs cDNA,RIKEN full-length enriched library, c  Mcm7 Adult male testis cDNA, RIKEN full-length enriched library, clone:492  Ezr;LOC100044177 Ezrin  Dpysl2 Dihydropyrimidinase- related protein 2  Stip1 Stress- induced-phosphoprotein 1  Trap1 Heat shock protein 75 kDa,  mitochondrial precursor  Gpd2 Glycerol phosphate dehydrogenase 2, Mitochondrial  Tkt Transketolase  Pcca Propionyl-CoA carboxylase alpha chain, mitochondrial precursor  Samhd1 Adult male cecum cDNA,  RIKEN full-length enriched library,  clone:91  Sars2 Seryl-tRNA synthetase,  mitochondrial precursor  Pkm2 Isoform M2 of Pyruvate  kinase isozymes M1/M2  Lgals3 19 kDa protein  Anxa2 Annexin A2  Vps29 16 kDa protein | anti-apoptotic molecular;  participates in cell adhesion.  binds to the epidermal growth factor receptor;  is required for normal nucleolar function in proliferating cells.  protects cell against oxidative stress by H(2)O(2).  negative regulation of cell proliferation; B cell activation.  participates in Cell Communication and structural molecule activity.  participates in Long-term depression, Tight junction and Wnt signaling pathway.  participates in Cell Communication and intermediate filament-based process.  enhances BCR signals and contributes to BCR-induced apoptosis.  ATP binding, catalytic activity, creatine kinase activity and nucleotide binding.  hnRNP K augments efficiency of VEGF mRNA translation stimulated by ANG II.  participates in Oxidative phosphorylation.  participates in cell adhesion, protein complex assembly and signal transduction.  a novel angiogenic factor that induces VEGF production through the HIF-1 pathway and induces migration.  adherens junction, cell communication, leukocyte transendothelial migration, regulation of actin cytoskeleton and tight junction.  participates in cell proliferation, DNA unwinding during replication, cell cycle, DNA replication, DNA replication initiation and regulation of transcription.  involved in connections of major cytoskeletal structures to the plasma membrane.  participates in cell differentiation and hydrolase activity.  participates in response to stress and rescues cells from apoptosis.  an anti-apoptotic molecule (anti-formation of ROS), induces MMP-9 expression in muscle cells.  regulates metabolism under anoxic conditions.  inhibits mast cell-dependent allergic reactions and inflammatory cytokines secretion.  ATP binding, biotin binding, catalytic activity, ligase activity, propionyl-CoA carboxylase activity  participates in immune response;  owns catalytic activity and hydrolase activity.  ATP binding, serine-tRNA ligase activity, nucleotide binding aminoacyl-tRNA ligase activity  interacts and cooperates with Oct-4 in regulating transcription.  regulates cell migration and adhesion to collagen-I and -IV, modulate inflammatory and immune responses, an anti-apoptotic molecule ( Enhances Akt activation and suppresses ERK Activation).  regulates angiogenesis, collagen fibril organization, fibrinolysis;  regulates stem cell adhesion, homing and engraftment following transplantation;  microbial clearance, establishment of inflammation and immune response.  participates in protein transport and owns hydrolase activity. |

Protein description: the name of each matched protein in the Swiss-Prot or Trembl database. Serial number: IPI accession number of EBI database. Sequence coverage: percent of identified sequence to the complete sequence of known protein. Ions score was -10×Log (P), where P was the probability that the observed match was a random event. Protein scores were greater than 60 were significant (p<0.05). The overall trend of spot “down/up” means that this protein spots were down-regulated or up-regulated in the primary MSCs as compared to the 5^th^ passaged MSCs (Variance multiple>5). PI value: theoretical isoelectric point of the matched protein. Nominal mass: theoretical molecular weight of the matched proteins in Da.
